# Relationship between family background and self-efficacy in adolescent table tennis players: a moderated mediation model

**DOI:** 10.3389/fpsyg.2023.1125493

**Published:** 2023-06-05

**Authors:** Ke He, Weiming Li, Zihao Li

**Affiliations:** ^1^Sports Coaching College, Beijing Sport University, Beijing, China; ^2^Institute of Physical Education and Training, Capital University of Physical Education and Sports, Beijing, China

**Keywords:** adolescent table tennis players, family background, self-efficacy, gender, training years

## Abstract

**Introduction:**

A moderated mediation model was constructed in this study to clarify the relationship between family background and self-efficacy of adolescent table tennis players, focusing on the mediating effect of technical learning engagement in the relationship as well as the moderating role of factors such as gender and training years.

**Methods:**

189 adolescent table tennis players (age: 13.69±1.28 years) were investigated as subjects using a questionnaire method.

**Results:**

(1) Family background, technical learning engagement, and self-efficacy were significantly and positively correlated (*p*<0.01), with girls’ technical learning engagement (M_female_=5.81, M_male_=5.19, *p*<0.01) and self-efficacy (M_female_=3.34, M_male_=2.66, *p*<0.01) significantly higher than boys’; (2) Technical learning engagement partially mediated the effect of family background on self-efficacy (ab=0.10, boot SE=0.02,95% CI=[0.07, 0.14]); (3) The first half of technical learning engagement’s mediating role was moderated by gender (B=0.05, *p*<0.01), with a more significant influence of family background on boys’ (B=0.24, *p*<0.001, 95% CI=[0.22, 0.26]) technical learning engagement than girls’ (B=0.19, *p*<0.001, 95% CI=[0.17, 0.21]); (4) The second half of technical learning engagement’s mediating role was moderated by training years (B=–0.21, *p*<0.001), with a more significant influence of technical learning engagement on the self-efficacy of adolescents with fewer training years (B=0.54, *p*<0.001, 95% CI=[0.39, 0.68]). The positive effect of technical learning engagement on self-efficacy gradually diminished with increasing training years, and the moderating effect of training years disappeared when the training years reached 8.94 years.

**Conclusion:**

(1) More attention should be paid to adolescent table tennis players with poor family backgrounds, who are more likely to have low self-efficacy. (2) Parents should never neglect their initiative in providing guidance and support to adolescent players involved in long-term professional table tennis training, especially for boys. (3) Coaches should pay close attention to the level of technical learning engagement of players with long training years, who are more likely to have lower self-efficacy as a result of their own emotional experiences, stagnant performance, etc.

## 1. Introduction

Competitive sports are at the core of global sports development, and China’s competitive sports are currently rising in the world with rapid development and impressive accomplishments. Table tennis has dominated the international arena for over half a century as the most popular sport in China (Xu et al., 2016; Zhang and Zhou, 2019). These achievements would not have been possible without the support of China’s national sports system and the cultivation of generations of young table tennis talents. Given the country’s unique historical context and national conditions, China’s competitive sports reserve talents primarily follow a tradition of intense training under the trinitarian principles of difficulty, rigor, and practicality. Athletes are constantly exposed to overload training and the challenge of physiological limits, especially adolescent players who undergo a period of psychological and physiological development and personality formation. As a result, adolescent players are prone to injury and disease, tend to passively accept the training contents assigned by coaches, and may even develop a dislike for and resistance to training, eventually lowering their sense of efficacy in training and jeopardizing athletic performance. As the most fundamental unit of human life, the family is a crucial setting that impacts an individual’s growth and development. Adolescents are at a critical juncture in their formation of self-awareness and thinking maturity, and supportive family background has a significant impact on their physical and mental well-being (Kleszczewska et al., 2019) as well as their cognitive development (Belen Barreto et al., 2017). Therefore, it is crucial to consider how self-efficacy can be safeguarded and enhanced in adolescent athletes from the perspective of family background.

Self-efficacy is a core concept of social cognitive theory, which refers to how confident individuals feel in their ability to use the skills they possess to perform a task (Bandura, 1986). It can affect the way individuals attribute things (Yeo and Tan, 2012) and motivate individuals to adopt positive behaviors (Carron et al., 1996; Ouweneel et al., 2011) and attitudes (Graydon, 1997; Judge and Bono, 2001) in a given task. Additionally, self-efficacy plays a positive role in goal setting (Cheng and Chiou, 2010), action orientation (Wolf et al., 2018), task performance (Barling and Abel, 1983; Pajares and Miller, 1994), academic achievement (Hwang et al., 2016), work engagement (Chase et al., 1994; Tan and Chou, 2018), and career exploration (Scott and Ciani, 2008). Most existing studies have explored the mediating effects of self-efficacy on the behavioral performance of the whole group of adolescents from the perspectives of family environment (Davis-Kean, 2005) and family socioeconomic status (Hsieh and Huang, 2014; Wiederkehr et al., 2015). The level of commitment and self-discipline in learning varies by gender due to family economic status, home environment, and parental educational intentions (Zimmerman and Martinez-Pons, 1990). Skill acquisition in table tennis is a practical process of mind–body unity that fosters the co-development of physical and cognitive activities. Adolescents go through complex mental activities at the same time when training and learning, including a constant state of competition, collaboration, overcoming, and performance change. The longer a student trains, the more their sense of experience with learning to train varies, which has varied degrees of impact on their self-efficacy (Richardson and Newby, 2006). In addition, in terms of economic capital, social capital, and the family environment, families with poor backgrounds are more likely to experience stress and uncertainty, which increases the likelihood of issues such as powerlessness, learned helplessness, and low self-esteem for adolescents (McLoyd, 1998) and diminishes their self-efficacy.

In terms of self-efficacy theory, this study focuses on the processes and mechanisms that influence the self-efficacy of adolescent table tennis players in terms of their family background, analyzes the mediating role of technical learning inputs and the moderating role of gender and training years, and makes two major theoretical contributions. First, this study sheds light on the mechanisms that influence self-efficacy in the training process of adolescent table tennis players from the perspective of family background, which enriches the theory of self-efficacy in the training process of adolescent table tennis players. Second, taking China, the world’s dominant table tennis player, as an example, the study of this group of Chinese adolescent table tennis players is of great relevance for future adolescent table tennis training in China and abroad. Therefore, the following objectives were set for this study: (1) to investigate the relationship between family background and self-efficacy of youth table tennis players, as well as the influencing mechanisms; (2) to investigate the mediating role of technical learning engagement of youth table tennis players; (3) to explore the moderating effects of gender and years of training factors in the various segments of the mediating role of technological learning inputs.

## 2. Literature review

### 2.1. Family background and self-efficacy

Recent research has revealed that family background characteristics, such as family structure, socioeconomic status, parental relationship quality, and parental desires, have an impact on the development of self-efficacy (Astone and McLanahan, 1991; Hsieh and Huang, 2014; Weisskirch, 2018). As early as the 1980s, Whitbeck (1987) put forth the hypothesis that adolescents’ self-efficacy would be directly or indirectly influenced by parental behaviors in the family context. Subsequently, scholars have argued about the effects of factors such as family economic status, family environment, and parental educational intentions on adolescent self-efficacy. For instance, Matthews and Gallo (2011) argued that families with a high socioeconomic level were more likely to offer their children better academic and material conditions, which had a positive impact on their children’s thinking, academic performance, and sense of efficacy. Adolescents were inspired to engage in similar habits and foster their sense of efficacy by observing their parents’ success in specific areas (Bandura, 2012). Furthermore, adolescents’ academic self-perceptions, expectations, and perceptions of task difficulty were found to be related to their parents’ expectations (Parsons et al., 1982). Parents can help children grow up with high expectations of themselves by being willing to act and verbally expressing their expectations. Based on the above findings, the first hypothesis proposed in this study is as follows:

*H1*: Family background positively predicts the self-efficacy in Chinese adolescent table tennis players.

### 2.2. Mediating role of technical learning engagement

Family background is closely related to learning engagement. It is found that adolescents with poor family backgrounds frequently experience more intra-family conflicts and less family warmth as a result of their parents’ lower socioeconomic status and less favorable family environment, which makes it more difficult for them to have a positive attitude toward learning (Terenzini et al., 2001; Randolph et al., 2006). This phenomenon is further corroborated by empirical studies demonstrating that parents with lower socioeconomic status have more negative parenting styles, such as paying less attention and showing insufficient affection and understanding to their children (Bae and Wickrama, 2015). In contrast, families with stronger parental educational aspirations are more likely to produce children with more educational attainments and academic achievements (Catsambis, 2001). Thus, family background is an important factor affecting adolescents’ technical learning engagement.

At the same time, technical learning engagement has a positive effect on individuals’ self-efficacy. According to social cognitive theory, learning is regulated and influenced by individual behavioral and situational factors (Shute, 2008). Students regulate their learning process based on cognition, learning engagement, and internal motivation (Panadero et al., 2018). Learning engagement is an important variable in the learning process, which assesses students’ engagement in learning activities during their studies (Jurik et al., 2014). A higher level of learning engagement has a positive impact on students’ academic achievements (Dresel and Haugwitz, 2008; Pat-El et al., 2012). Accordingly, the second hypothesis proposed in this study is as follows:

*H2*: Family background influences the self-efficacy of Chinese adolescent table tennis players through technical learning engagement.

### 2.3. Moderating effect of gender

The impact of different family backgrounds on children exhibits gender differences in many aspects. For example, families with lower socioeconomic status see investing in their children’s education as an important strategy for family well-being. The resource dilution model presupposes that there is a cap on the resources available to the family and that the resources allotted to each child decline as the birth rate rises (Blake, 1981). However, the degree of this loss differs significantly between boys and girls. From the perspective of Western academics, families should invest more in daughters when they are in less fortunate circumstances (Trivers and Willard, 1973), while there is still a “preference for sons over daughters” in some Chinese families due to China’s patriarchal culture. Boys’ education is more important to parents in rural China (Hannum, 2003), where the family’s declining economic status is more likely to have a detrimental impact on girls’ education (Hannum, 2005). Gender inequality in education tends to decline as the socioeconomic status of families increases (Yeung, 2013).

Previous studies have demonstrated that girls exhibit higher levels of engagement and self-regulation in learning compared to boys and that girls outperform boys in planning, goal setting, structuring, and self-monitoring in learning (Zimmerman and Martinez-Pons, 1990). Studies have revealed that when it comes to learning tactics and usage, girls tend to be more organized, allocate their study time appropriately, and are able to devote more metacognition to their learning (Ruffing et al., 2015). Girls tend to be more engaged and self-regulated in their learning than boys, especially as they enter adolescence (Klimstra et al., 2009). Due to these gender differences, boys may be more dependent than girls on the influence of factors and structures within the family for learning engagement. Accordingly, the third hypothesis proposed in this study is as follows:

*H3*: The mediating role of the first half of the technical learning engagement is moderated by gender, with family background having a more significant impact on technical learning engagement in boys.

### 2.4. Moderating effect of training years

In academic settings, students’ learning engagement refers to the quality of effort they put into achieving desired outcomes, such as good grades (Hu and Kuh, 2002; Richardson et al., 2004). Previous research has illustrated a positive correlation between students’ learning engagement and motivational factors (Kanuka, 2005), learning factors (Whipp and Chiarelli, 2004), and emotional experiences (Usan Supervia and Quilez Robres, 2021). However, limited research has been conducted to empirically investigate the relationship between students’ learning engagement and motivational and learning variables (interest, self-efficacy, and self-regulation). For instance, Bates and Khasawneh (2007) found that higher computer self-efficacy can be observed in students who spent more time using online learning technologies and were more engaged in the learning process. Sporting skill acquisition is a long-term process, and students’ emotional experiences decrease as they train for a longer time (Richardson and Newby, 2006). Long-term follow-up studies in schools have shown that positive emotional experiences lead to higher levels of learning engagement and promote positive changes in coping styles, which in turn promotes students’ self-efficacy (Dong et al., 2020). Accordingly, the fourth hypothesis proposed in this study is as follows:

*H4*: The mediating role of the second half of the technical learning engagement is moderated by training years. The positive effect of technical learning engagement on self-efficacy decreases as the number of training years increases.

**Figure 1:**
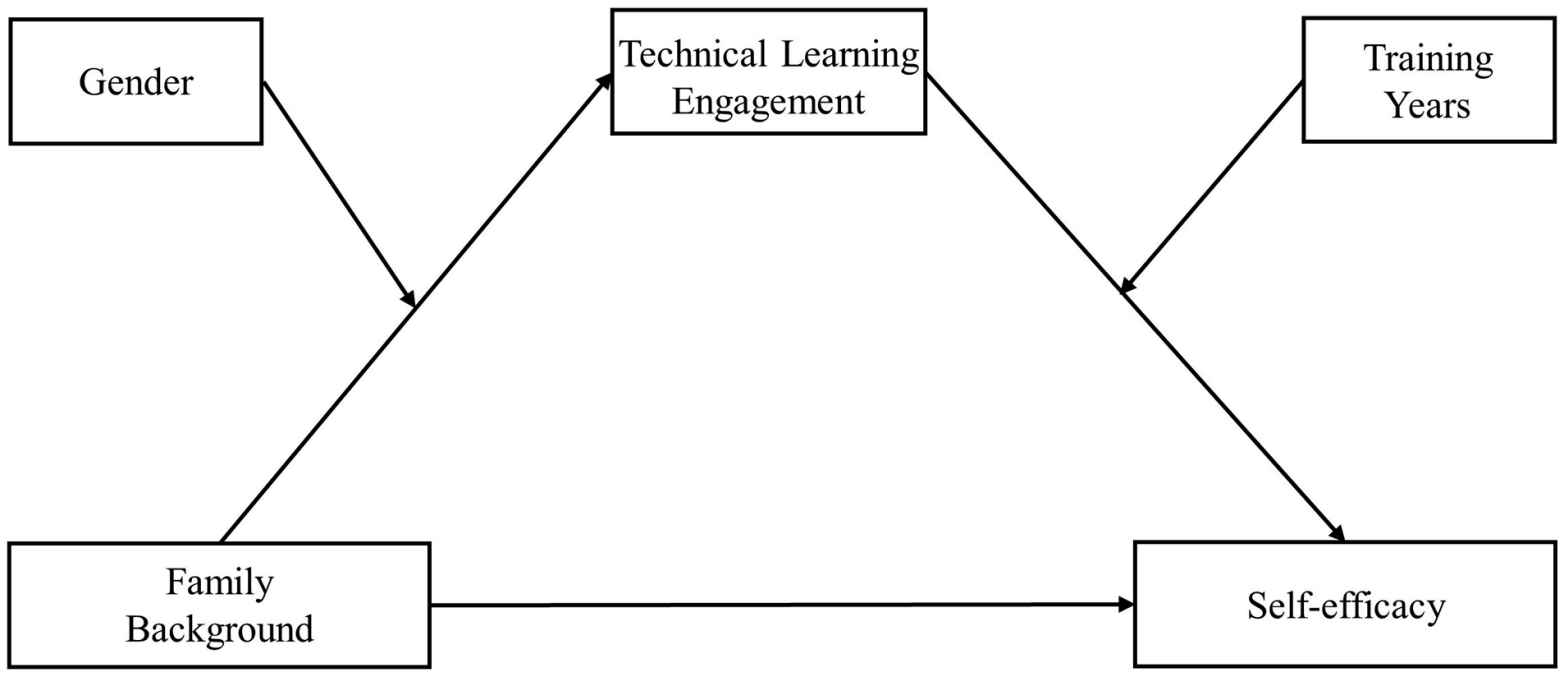
Hypothetical model of the mediating role of technological learning engagement and the moderating role of gender and training years.

In summary, this study proposed that the technical learning engagement of adolescent table tennis players might play a mediating role in the relationship between family background and self-efficacy, and that the factors of gender and training years have moderating effects on the first and second halves of the mediation model, respectively. The proposed model is shown in Figure 1.

## 3. Methods

### 3.1. Data collection

The cluster sampling method was utilized in this study to conduct a group administration on adolescent table tennis players from Shichahai and Haidian sports schools in Beijing. Our study was approved by the ethical committee of the Capital University of Physical Education And Sports (2022A75). The questionnaires were completed and collected on the spot. 189 questionnaires were distributed successfully according to the inclusion criteria of more than 5 years of participation in table tennis training with a frequency of more than 3 times a week. Additionally, questionnaires with unanswered questions or the same answers to 8 or more consecutive questions were considered invalid and excluded. 181 valid questionnaires were finally collected with an efficiency of 95.77%. The subjects ranged in age between 11 and 16 years old (*M* = 13.69, SD = 1.28), with the majority of training years lasting between 5 and 10 years (*M* = 7.83, SD = 1.54). There was no significant difference in the gender distribution in terms of school type (*χ*^2^ = 2.88, *p* = 0.09); there was no gender difference in the age distribution (*M*_female_ = 13.72, *M*_male_ = 13.65, *t* = −3.44, *p* = 0.73) and school type (*M*_Shichahai_ = 13.59, *M*_Haidian_ = 13.75, *t* = −0.83, *p* = 0.41).

### 3.2. Data measurement

#### 3.2.1. Family background

This study measures family background in terms of two dimensions: family socioeconomic background and family cultural environment. The family socioeconomic background dimension was synthesized using two variables: “family income” and “parents’ education level” (Bradley and Corwyn, 2002; Marks and Mooi-Reci, 2015). The academic accomplishment of earlier generations (Bourdieu and Passeron, 1977) and the supportive attitudes of family members (Zimdars et al., 2009; Jæger, 2011) are commonly used to assess the family cultural environment dimension. Since this study was conducted in a professional-technical setting, the level of family cultural environment was synthesized using three variables: “number of sports-related jobs in the family,” “parents’ attitudes toward their children’s long-term table tennis training,” and “parents’ attitudes toward their children becoming professional table tennis players” (Guo and Min, 2006).

The family income was denoted by numbers 1 to 5 for less than 5,000 yuan, 5,000 ~ 15,000 yuan, 15,000 ~ 30,000 yuan, 30,000 ~ 60,000 yuan, and more than 60,000 yuan, respectively (Xia, 2022). The education level of parents was denoted by numbers 1 to 5 for the groups of elementary school and below, junior high school (including junior high school without a degree), high school or junior college (including high school without a degree), college (including night college and electric college), undergraduate and above, respectively. The number of persons engaged in sports-related work in the household was denoted by numbers 1 to 5 for the groups of 1 or less, 2, 3, 4, 5 or more, respectively. The numbers 1 to 5 were used to represent parents’ attitudes toward their children’s long-term table tennis training and becoming professional table tennis players, including very opposed, opposed, average, very supportive, and somewhat supportive, respectively. The questionnaire consisted of 5 items and was scored on a 5-point Likert scale. The scores were synthesized by first standardizing and summing the scores for each dimension variable to obtain scores for both the family socioeconomic background dimension and the family cultural environment dimension. Finally, the standardized scores of both dimensions were summed to obtain the family background score. The higher the score, the higher the family background. The Cronbach’s α coefficient of the questionnaire in the actual test was 0.76.

#### 3.2.2. Technical learning engagement

The UtrechtWork Engagement Scale-student (UWES-S) developed by Schaufeli et al. (2002) was used to measure students’ engagement in technical learning. The UWES-S has been widely used by researchers and demonstrates desirable reliability and validity (Moon and Ke, 2020; Wang et al., 2021; Li et al., 2022). In particular, the questionnaire has been translated and adapted to use among Chinese populations (Fong and Ng, 2012). Since this study was conducted in a professional-technical setting, the original scale was partially revised by including a sample question like “I can recover quickly from mental fatigue during technical learning.” The Learning Engagement Scale consisted of 17 items and was graded on a 7-point Likert scale, ranging from 1 denoting “never” to 7 denoting “always.” The total scale was established from 3 dimensions: motivation, energy, and concentration. Higher scores indicate a greater commitment to learning. The factors and overall Cronbach’s α coefficients for this scale in the actual test were 0.79, 0.81, 0.72, and 0.91, respectively. The corresponding results of confirmatory factor analysis were: *χ*^2^/df = 1.14 (df = 116), RMSEA = 0.03, IFI = 0.99, TLI = 0.98, and CFI = 0.98.

#### 3.2.3. Self-efficacy

The General Self-Efficacy Scale (GSES) developed by Schwarzer et al. (1997) was used to measure students’ self-efficacy. The GSES has been widely used by researchers and demonstrates desirable reliability and validity (Luszczynska et al., 2005; Azizli et al., 2015; Lazić et al., 2021). The questionnaire has been culturally appropriate for the Chinese context (Sun et al., 2021). This 10-item scale is scored on a 4-point Likert scale to measure the individual’s self-efficacy in the face of frustration or difficulty, ranging from 1 denoting “not at all true” to 4 denoting “completely true,” with higher scores representing higher self-efficacy. The Cronbach’s α coefficient of the scale in the actual test was 0.94, and the corresponding results of validation factor analysis were: *χ*^2^/df = 0.67 (df = 35), RMSEA = 0, NFI = 0.98, RFI = 0. 98, and CFI = 1.

### 3.3. Data processing

SPSS 27.0 was used for data processing, and GraphPad Prism 9 was used for producing moderating effect plots in this study. First, Harman’s single-factor test was employed for common method bias. Second, descriptive statistics were used to demonstrate the current status of various variables in adolescent table tennis players; Pearson correlation was used to reflect the relationship between the variables. Third, the mediating role of technical learning inputs was investigated using Model 4 in the SPSS macroprogram PROCESS (PROCESS is a computational aid in the form of a freely available macro for SPSS and SAS) (Hayes, 2018). Finally, Model 21 in PROCESS was used to test the moderating effects of the gender and training years in the first and second halves of the mediation model, respectively. A bootstrap method (5,000 bootstrap samples) with 95% confidence intervals (CI) was used to test the significance of the effects during the study (MacKinnon et al., 2004; Fritz and MacKinnon, 2007). To avoid multiple correlations, all observed variables were standardized for z-scores before analyzing Model 4 and Model 21.

## 4. Results

### 4.1. Control and test of common method bias

In this study, anonymous questionnaire survey and reverse presentation were used for some items to procedurally control any potential common bias. The collected data were tested for common method bias using Harman’s single-factor test. The results of the unrotated exploratory factor analysis extracted a total of five factors with characteristic roots greater than one, with a maximum factor variance explained as 38.83% (less than 40%), indicating that there was no significant common method bias.

### 4.2. Descriptive statistics and correlation analysis

Table 1 displays the means, standard deviations, and correlation coefficients for each variable. The results of the correlation analysis revealed that there were highly positive correlations between training years, family background, technical learning engagement, and self-efficacy, all of which reached an extremely significant level (*p* < 0.01). Female athletes scored considerably higher than male athletes on the overall family background score (*M*_female_ = 2.88, *M*_male_ = −1.50, *t* = −8.00, *p* < 0.01), technical learning engagement (*M*_female_ = 5.81, *M*_male_ = 5.19, *t* = −6.97, *p* < 0.01), and self-efficacy (*M*_female_ = 3.34, *M*_male_ = 2.66, *t* = −7.99, *p* < 0.01).

**Table 1:** Results of descriptive statistics, correlation analysis between study variables.

Variable (*N* = 181)	*M*	*SD*	1	2	3	4
1. Training years (years)	7.83	1.54	1			
2. Family background	0.00	4.26	0.79**	1		
3. Technical learning engagement	5.51	0.67	0.76**	0.93**	1	
4. Self-efficacy	3.00	0.66	0.67**	0.91**	0.91**	1

** *p* < 0.01.

### 4.3. Direct effect of technical learning engagement on self-efficacy

The mediation effect of technical learning engagement between family background and self-efficacy was examined using Model 4 in PROCESS developed by Hayes (2013), with age, gender, and training years controlled. As shown in Tables 2, 3, family background significantly and positively predicted self-efficacy (*B* = 0.15, *t* = 19.37, *p* < 0.001). Moreover, technical learning engagement significantly and positively predicted self-efficacy (*B* = 0.33, *t* = 6.43, *p* < 0.001). The bias-corrected Bootstrap test indicated a significant mediating effect of technical learning engagement with an indirect effect value of 0.10, a 95% confidence interval of [0.07, 0.14], and a mediating effect of 43.48% of the total effect (0.23). This implies that family background can have both a direct effect and a partial mediating effect on self-efficacy through technical learning engagement.

**Table 2:** Mediation model test for technical learning engagement.

Regression (*N* = 181)		Overall fitted index			Significance of regression coefficients	
Result variables	Predictive variables	*R*	*R^2^*	*F (df)*	*B*	*t*
Self-efficacy		0.92	0.84	236.36^***^		
	Age				0.07	3.26^**^
	Gender				−0.10	−2.24^*^
	Training years				−0.10	−3.19^**^
	Family background				0.15	19.37^***^
Technical learning engagement		0.94	0.88	314.00^***^		
	Age				0.04	2.19^*^
	Gender				0.03	0.81
	Training years				0.03	1.07
	Family background				0.14	20.15^***^
Self-efficacy		0.93	0.87	240.62^***^		
	Age				0.05	2.52^*^
	Gender				−0.12	−2.86^**^
	Training years				−0.12	−4.04^***^
	Technical learning engagement				0.33	6.43^***^
	Family background				0.08	6.44^***^

Gender was dummy coded in the model: 1 = male, 0 = female; each continuous variable was standardized and brought into the regression equation; *p < 0.05, **p < 0.01, ***p < 0.001.

**Table 3:** Decomposition of total effect, direct effect and mediating effect.

	Effect	Boot SE	Boot LLCI	Boot ULCI	Relative effect value
Total effect	0.23	0.08	0.14	0.17	
Direct effect	0.13	0.02	0.08	0.16	56.52%
Mediating effect	0.10	0.02	0.07	0.14	43.48%

Boot SE, Boot LLCI, and Boot ULCI refer to the standard error, lower and upper limits of the 95% confidence interval of the indirect effects estimated by the bias-corrected percentile Bootstrap method, respectively; all values are rounded to two decimal places.

### 4.4. Test of moderated mediating effect

Model 21 in PROCESS was used to test the moderated mediation model with age controlled to examine the moderating effects of factors such as gender and training years in the first and second halves of the mediation model for analyzing the impact of family background on adolescent table tennis players’ self-efficacy through technical learning engagement (Model 21 assumes that the first and second halves of the mediation model are moderated, consistent with the theoretical model in this study). The results in Table 4 illustrated that after introducing factors of gender and training years to the model, the product term of family background and gender was a significant predictor of technical learning engagement (*B* = 0.05, *t* = 3.28, *p* < 0.01), and the product term of technical learning engagement and training years was a significant predictor of self-efficacy (*B* = -0.21, *t* = −5.57, *p* < 0.001). Based on this, it can be inferred that gender moderates the prediction of technical learning engagement by family background, and training years moderate the prediction of self-efficacy by technical learning engagement.

**Table 4:** Mediated model tests with moderation.

Regression (*N* = 181)		Overall fitted index			Significance of regression coefficients	
Result variables	Predictive variables	*R*	*R^2^*	*F (df)*	*B*	*t*
Technical learning engagement		0.94	0.88	333.47^***^		
	Age				0.06	2.39^*^
	Gender				−0.02	−0.24
	Family background				0.19	16.66^***^
	Family background*gender				0.05	3.28^**^
Self-efficacy		0.94	0.89	274.91^***^		
	Age				0.04	1.85
	Family background				0.16	8.78^***^
	Training years				−0.19	−4.41^***^
	Technical learning engagement				0.33	4.26^***^
	Technical learning engagement*training years				−0.21	−5.57^***^

Gender was dummy coded in the model: 1 = male, 0 = female; each continuous variable was standardized and brought into the regression equation； *p < 0.05, **p < 0.01, ***p < 0.001.

**Figure 2:**
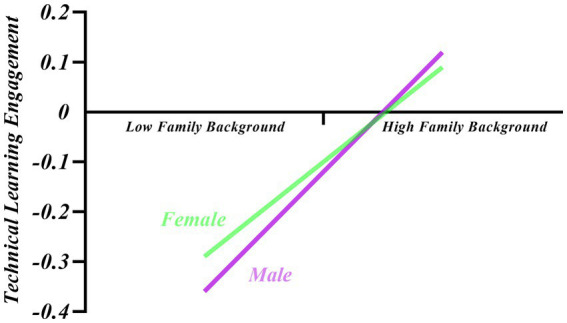
The moderating role of gender in the relationship between family background and technology learning engagement.

Family background exhibited a greater effect on boys’ technical learning engagement compared to girls, with a judgment index of −0.01 and a confidence interval of [−0.017,-0.004] (excluding 0), indicating a significant moderating effect of gender on family background and technical learning engagement. Separate analyses were conducted for male and female subjects to better understand the essence of the interaction between family background and gender. The results illustrated that the values of the mediating effect and the 95% Bootstrap confidence intervals were significantly different for the two groups, as shown in Table 5. According to Figure 2, further simple slope analysis revealed that family background had a significant positive predictive effect on technical learning engagement for both male and female adolescents, which was higher for male adolescents (simple slope = 0.24, *t* = 26.72, *p* < 0.001) than for female adolescents (simple slope = 0.19, *t* = 16.66, *p* < 0.001).

**Table 5:** Mediating effects of technical learning engagement for subjects of different genders.

Mediator variables	Gender	Effect	Boot SE	Boot LLCI	Boot ULCI
Technical learning engagement	Male	0.24	0.01	0.22	0.26
	Female	0.19	0.01	0.17	0.21

**Figure 3:**
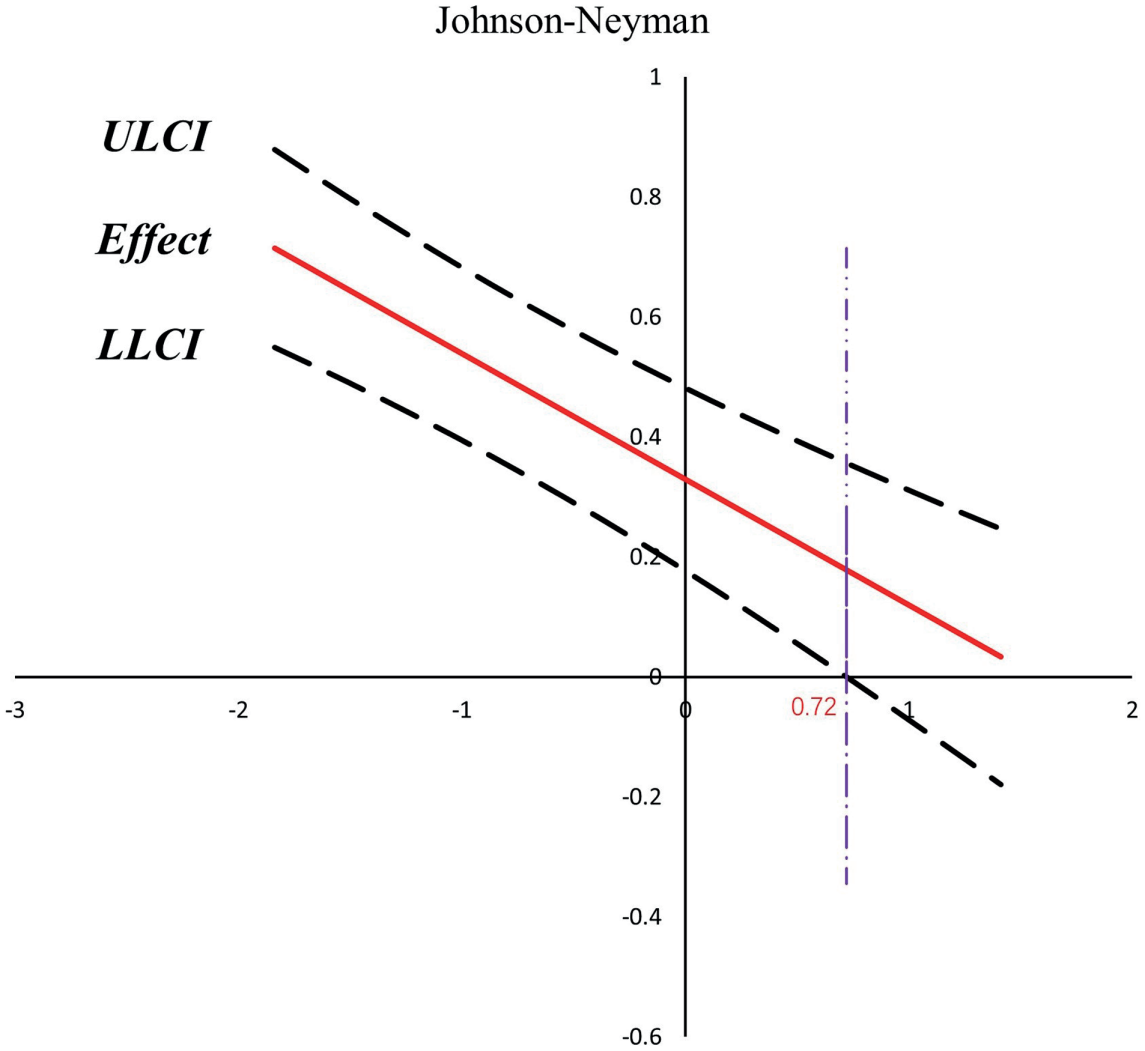
Johnson-Neyman diagram of the moderating effect of training years. ULCI refers to upper limit of the 95% confidence interval; LLCI refers to the lower limit of the 95% confidence interval.

**Figure 4:**
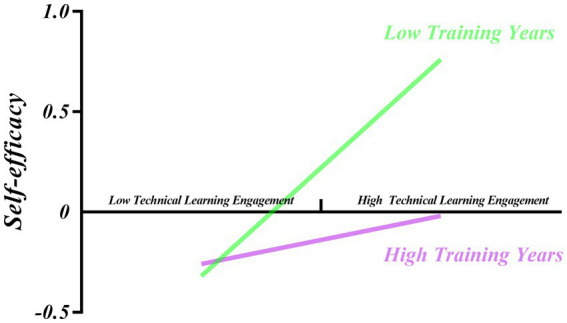
Moderating role of training years in the relationship between technical learning engagement and self-efficacy.

Based on the findings above, it is evident that different training years have a moderating effect in the second half of the model for analyzing the impact of family background on self-efficacy through technical learning engagement. To further understand the interaction between technical learning engagement and training years, data from different training years were divided into the high training years group (M + 1SD) and the low training years group (M-1SD) for analysis, as shown in Table 6. There was a significant difference in the impact of technical learning engagement on self-efficacy in the group with low training years and no significant difference in the group with high training years. Further visualization is shown in Figure 3, where the moderating effect is always greater than 0, implying that technical learning engagement has a positive mediation effect on self-efficacy regardless of the moderating effect of training years. As training years increase, the positive moderating effect of technical learning engagement on self-efficacy gradually decreases. There is no significant effect of technical learning engagement on self-efficacy until the training years reach the critical value of 0.72 (obtained by standardizing the value of 8.94 years, at which the moderating effect of training years disappeared). According to Figure 4, the regression coefficient was significantly larger in the low training years group (simple slope = 0.54, *t* = 7.37, *p* < 0.001) than in the high training years group (simple slope = 0.12, *t* = 1.24, *p* = 0.22), according to a simple slope analysis (Figure 4). That is, the effect of technical learning engagement on self-efficacy was higher when training years were short. When training years were long, the impact factor of technical learning engagement on self-efficacy decreased as training years increased. Furthermore, training years had a significant negative moderating effect on the relationship between technical learning engagement and self-efficacy, diminishing the positive effect of technical learning engagement on self-efficacy until the moderating effect disappeared.

**Table 6:** Mediating effects of technical learning engagement of subjects with different training years.

Mediator variables	Training Years (years)	Effect	Boot SE	Boot LLCI	Boot ULCI
Technical learning engagement	6.29 (M-1SD)	0.54	0.07	0.39	0.68
	7.83 (M)	0.33	0.08	0.18	0.48
	9.37 (M + 1SD)	0.12	0.10	−0.07	0.31

## 5. Discussions

A moderated mediation model was constructed in this study to clarify the relationship between family background and self-efficacy of adolescent table tennis players, focusing on the mediating effect of technical learning engagement in the relationship, as well as the moderating role of factors such as gender and training years. The results revealed that (1) family background, technical learning engagement, and self-efficacy were significantly and positively correlated (*p* < 0.01), with girls’ technical learning engagement (M_female_ = 5.81, M_male_ = 5.19, *p* < 0.01) and self-efficacy (M_female_ = 3.34, M_male_ = 2.66, *p* < 0.01) significantly higher than boys’; (2) technical learning engagement partially mediated the effect of family background on self-efficacy [ab = 0.10, boot SE = 0.02,95% CI = (0.07, 0.14)]; (3) the first half of technical learning engagement’s mediating role was moderated by gender (*B* = 0.05, *p* < 0.01), with a more significant influence of family background on boys’ [*B* = 0.24, *p* < 0.001, 95% CI = (0.22, 0.26)] technical learning engagement than girls’ [*B* = 0.19, *p* < 0.001, 95% CI = (0.17, 0.21)]; (4) the second half of technical learning engagement’s mediating role was moderated by training years (*B* = -0.21, *p* < 0.001), with a more significant influence of technical learning engagement on the self-efficacy of adolescents with fewer training years [*B* = 0.54, *p* < 0.001, 95% CI = (0.39, 0.68)]. The positive effect of technical learning engagement on self-efficacy gradually diminished with increasing training years, and the moderating effect of training years disappeared when the training years reached 8.94 years.

The correlation analysis revealed a significant positive relationship between family background and self-efficacy of adolescent table tennis players. The higher the overall score of adolescent players’ family background, the higher their self-efficacy. This outcome reaffirms that factors related to family backgrounds, such as family economic status, family environment, and parental educational intentions, have a direct and indirect impact on adolescent players’ self-efficacy (Astone and McLanahan, 1991; Hsieh and Huang, 2014; Weisskirch, 2018). Furthermore, there is a positive correlation between family background and adolescents’ technical learning engagement, with better family backgrounds associated with higher technical learning engagement, which is consistent with theoretical predictions. Adolescents with high-level family backgrounds are more likely to have better academic and material conditions due to their family’s higher socioeconomic status (Matthews and Gallo, 2011). They may also be motivated to engage in similar behaviors by their parents’ role modeling in their field of expertise and their willingness to set high expectations for their technical learning achievements and language (Catsambis, 2001; Bandura, 2012). Additionally, this study discovered a substantial positive association between self-efficacy and technical learning engagement. Adolescent players’ technical learning in table tennis is greatly influenced by factors such as training, competition, and external motivational support such as family support, recognition from others, and athletic achievement. They gradually lose their ability to experience and evaluate themselves if their commitment to technical learning decreases, which will lower their self-efficacy (Siu et al., 2014).

Further mediation analysis revealed that technical learning engagement mediated the relationship between family background and self-efficacy, and that family background influenced the technical learning engagement and self-efficacy of adolescent table tennis players. The results support previous research that adolescents with poor family backgrounds are more likely to experience increased intra-family conflicts and reduced family warmth due to their parents’ lower socioeconomic status and less favorable family environment, making it more difficult for them to engage in learning with a positive attitude and ultimately leading to their lower self-efficacy (Randolph et al., 2006; Pat-El et al., 2012). This finding allows us to refocus on family factors and technical learning engagement, rather than the earlier focus on aspects such as youth technical training and competitiveness, providing fresh ideas for improving the technical level and training efficacy of disadvantaged adolescent players. Specifically, parents with poor family backgrounds can enhance the technical learning engagement and self-efficacy of these adolescent table tennis players by adopting a more positive parenting style, such as showing more warmth and understanding to their children (Masarik and Conger, 2016). Second, youth training is regulated and influenced by the own behavioral and situational factors of adolescent table tennis players (Martin and Gill, 1991; Seidel, 2006; Shute, 2008). Therefore, coaches must focus not only on training and game performance but also on the development of contextual factors such as a positive training atmosphere and healthy competition (Psychountaki and Zervas, 2000) among players in order to enhance their sense of efficacy. As for future research, on the one hand, more importance should be attached to identifying additional mediators that may bridge the gap between family background and efficacy to fully reveal the pathways through which family background affects self-efficacy in adolescent table tennis players; on the other hand, research on other sports can be conducted to investigate the common patterns of family background influencing self-efficacy in the context of various sports characteristics.

Additionally, the effect of family background on the technical learning engagement of adolescent table tennis players was moderated by gender, with a more significant influence of family background on boys’ technical learning engagement and a stronger indirect effect of technical learning engagement on self-efficacy. This finding is a reflection of China’s distinctive patriarchal culture with a preference for boys (Chu et al., 2007), which differs from the findings of Trivers and Willard (1973) and others in a Western cultural context. Previous research has shown that the pattern of gender differences in academic self-efficacy varies across domains (Huang, 2013). Boys showed higher self-efficacy than girls in math, computers and social sciences (Chou, 2001; Peng et al., 2006). In contrast, girls had significantly higher levels of self-efficacy in self-regulated learning (Britner and Pajares, 2001), engagement in learning and self-regulation (Zimmerman and Martinez-Pons, 1990) than boys, which is consistent with the theoretical prediction of this study: male adolescent table tennis players perform poorly than female adolescent players in terms of planning, goal setting, and self-monitoring during training, thus with a lower level of learning engagement. As family backgrounds improve, boys tend to have better development and academic accomplishment due to more parental attention (Terenzini et al., 2001), more positive parenting (Randolph et al., 2006), and higher educational expectations (Bae and Wickrama, 2015). Therefore, it can be concluded that family background has a greater impact on the technical learning engagement of male adolescent table tennis players.

Finally, the effect of technical learning engagement on the self-efficacy of adolescent table tennis players was moderated by training years, with a more significant influence of technical learning engagement on the self-efficacy of players with fewer training years. The positive effect of technical learning engagement on self-efficacy gradually diminished as training years increased. This finding differs from that of Bates and Khasawneh (2007) in that the effort level of adolescents with fewer training years tended to be higher, leading to more noticeable performance gains, especially in less difficult technical tasks, which resulted in lower gains in self-efficacy (Treasure et al., 1996; Linnenbrink and Pintrich, 2003). However, the positive effect of technical learning engagement decreased with training years due to increased learning pressure and factors such as win-loss and competition among players. Players’ emotional experience will decline and ultimately lead to a decrease in self-efficacy (Richardson and Newby, 2006). Therefore, it can be concluded that technical learning engagement has a greater effect on self-efficacy in adolescent players with fewer training years, and this positive effect will gradually diminish as their training years increase.

## 6. Conclusion

This study analyzed the processes and mechanisms of the impact of family background on the self-efficacy of adolescent table tennis players, as well as the mediating role of technical learning engagement and the moderating effects of gender and training years. We found that family background had a predictive effect on the self-efficacy of adolescent table tennis players. The path was partially mediated by technical learning engagement. The mediating effect of technical learning engagement in the first half was moderated by gender, with a more significant influence of family background on boys’ technical learning engagement. The mediating effect of technical learning engagement in the second half was moderated by training years, with a more significant influence of technical learning engagement on the self-efficacy of adolescent players with fewer training years. The positive effect of technical learning engagement on self-efficacy gradually diminished as training years increased, and the moderating effect of training years disappeared at 8.94 years. This study still has some implications for enhancing the self-efficacy of adolescent table tennis players and promoting their skill acquisition and healthy development, both physically and mentally. First, more attention should be paid to adolescent table tennis players with poor family backgrounds, who are more likely to have low self-efficacy. Second, parents should never neglect their initiative in providing guidance and support to adolescent players involved in long-term professional table tennis training, especially for boys. Third, coaches should pay close attention to the level of technical learning engagement of players with long training years, who are more likely to have lower self-efficacy as a result of their own emotional experiences, stagnant performance, etc.

## 7. Limitations

It is essential to recognize the several limitations of the current study. First, the sample size of this study was relatively small, and the objects were all selected from Haidian and Shichahai Gymnasium in Beijing, China. A larger sample of adolescent table tennis players engaged in long-term professional training in more cities should be examined in future studies, taking into account the unique environment and cultural context of table tennis talent development in China. Second, the disparity in levels of competitiveness among adolescents from different sports schools was not considered when analyzing the effect of family background on the self-efficacy of adolescent table tennis players in this study. This factor may have different effects on adolescents involved in long-term professional training. Therefore, multilevel models should be employed in future studies to simulate the effects of different training levels. Third, the effects of various sub-dimensions of family background, which serve as composite variables in this study, can be further investigated, such as family economic status, family support, parental educational intentions, and parental praise and criticism.

## Data availability statement

The original contributions presented in the study are included in the article/Supplementary material, further inquiries can be directed to the corresponding author.

## Ethics statement

The studies involving human participants were reviewed and approved by the Capital University of Physical Education and Sports. Written informed consent to participate in this study was provided by the participants’ legal guardian/next of kin.

## Author contributions

KH and ZL contributed to the conception and design of the study. WL organized the database. KH and WL performed the statistical analysis. KH wrote the first draft of the manuscript. WL and ZL wrote sections of the manuscript. All authors contributed to the manuscript revision, read, and approved the submitted version.

## Conflict of interest

The authors declare that the research was conducted in the absence of any commercial or financial relationships that could be construed as a potential conflict of interest.

## Publisher’s note

All claims expressed in this article are solely those of the authors and do not necessarily represent those of their affiliated organizations, or those of the publisher, the editors and the reviewers. Any product that may be evaluated in this article, or claim that may be made by its manufacturer, is not guaranteed or endorsed by the publisher.
