# Supplementary material for: Relationship between family background and self-efficacy in adolescent table tennis players: a moderated mediation model
Source: Front Psychol. 2023 Jun 5;14:1125493. doi: 10.3389/fpsyg.2023.1125493 (PMC10283351; doi:10.3389/fpsyg.2023.1125493)
Supplement: Supplementary file 2 [file Data_Sheet_2.docx]

**Family background and self-efficacy of adolescent table tennis players questionnaire**

**Hello, we are conducting a survey on family background and self-efficacy in youth table tennis players to explore the relationship between family background and self-efficacy. We would appreciate your help in filling out this questionnaire with ten minutes of your time. This questionnaire is filled in anonymously, and all data are used for statistical analysis only, so please feel free to fill in. There are no right or wrong options for the questions, so please fill them out as you see fit. Thank you for your support!**

**I. Basic Information**

1、Your gender: A. Male B. Female

2、Your age is__________.

3、Your training years are__________.

4、Your daily training time is____________.

5、The number of times you train each week____________.

**II. Family background**

1. Your monthly family income is

A. Less than 5,000 yuan

B. 5,000~15,000 yuan

C. 15-30,000 Yuan

D. 30,000-60,000 Yuan

E. More than 60,000 yuan

2. The education level of your parents

A. Elementary school and below

B. Junior high school (including junior high school without a degree)

C. High school or junior college (including high school without a degree)

D. College (including night college and electric college)

E. Undergraduate and above

3. The number of persons engaged in sports-related work

A. 1 or less

B. 2 persons

C. 3 persons

D. 4 persons

E. 5 or more

4. Your parents' attitude towards your long-term table tennis training

A. Very opposed

B. Opposed

C. Average

D. Very supportive

E. Somewhat supportive

5. Your parents' attitude towards you becoming a professional table tennis player

A. Very opposed

B. Opposed

C. Average

D. Very supportive

E. Somewhat supportive

**Ⅲ. Technical learning engagement**

**Instructions:** **The following are the 17 items responding to the technological learning inputs, and the level of agreement with them is divided into 7 levels.** **The scores were** **1, 2, 3, 4, 5, 6 and 7 points for "Never", "Almost never", "Rarely", "Sometimes", "Often", "Very often", "Always", respectively.** **Please check one of the most appropriate answers based on your judgment of the level of agreement for each item.**

|  |  | **Never** | **Almost never** | **Rarely** | **Sometimes** | **Often** | **Very often** | **Always** |
| --- | --- | --- | --- | --- | --- | --- | --- | --- |
| 1 | I am energetic in the technical learning process. |  |  |  |  |  |  |  |
| 2 | I find table tennis training very valuable and meaningful. |  |  |  |  |  |  |  |
| 3 | During the technical learning process, I felt that the time passed quickly. |  |  |  |  |  |  |  |
| 4 | I am energetic during table tennis training or lessons. |  |  |  |  |  |  |  |
| 5 | I am interested in table tennis training. |  |  |  |  |  |  |  |
| 6 | The technical learning process was so focused that I forgot everything around me. |  |  |  |  |  |  |  |
| 7 | Table tennis training stimulates my intellectual curiosity. |  |  |  |  |  |  |  |
| 8 | As soon as I wake up in the morning, I am filled with the power of table tennis training. |  |  |  |  |  |  |  |
| 9 | I experience joy when I concentrate on my table tennis training. |  |  |  |  |  |  |  |
| 10 | I am satisfied with my table tennis training. |  |  |  |  |  |  |  |
| 11 | I concentrate on my table tennis training. |  |  |  |  |  |  |  |
| 12 | I forget everything around me during table tennis training. |  |  |  |  |  |  |  |
| 13 | I can energize continuous table tennis training for a long time. |  |  |  |  |  |  |  |
| 14 | I reached a state of forgetfulness during table tennis training. |  |  |  |  |  |  |  |
| 15 | I was able to recover quickly from the technical learning process, even if I was mentally exhausted. |  |  |  |  |  |  |  |
| 16 | I can concentrate during the technical learning process and am not easily distracted. |  |  |  |  |  |  |  |
| 17 | Even when table tennis training was not going well, I was able to keep going with energy. |  |  |  |  |  |  |  |

**Ⅳ. Self-efficacy**

**Instructions: The following is a list of 10 items that reflect self-efficacy, and the level of agreement is divided into 4 levels. The scores were 1, 2, 3 and 4 for "not at all correct", "somewhat correct", "mostly correct" and "completely correct" respectively.** **Please check one of the most appropriate answers based on your judgment of the level of agreement for each item.**

|  |  | **Not at all correct** | **Somewhat correct** | **Mostly correct** | **Completely correct** |
| --- | --- | --- | --- | --- | --- |
| 1 | I can always solve problems if I do my best. |  |  |  |  |
| 2 | Even if others oppose me, I still have the means to get what I want. |  |  |  |  |
| 3 | It is easy for me to stick to my ideals and reach my goals. |  |  |  |  |
| 4 | I am confident that I can effectively handle anything that comes my way. |  |  |  |  |
| 5 | With my talent, I will be able to handle unexpected situations. |  |  |  |  |
| 6 | If I put in the necessary effort, I will be able to solve most of the puzzles. |  |  |  |  |
| 7 | I can face difficulties calmly because I trust my ability to handle them. |  |  |  |  |
| 8 | When faced with a difficult problem, I can usually find several solutions. |  |  |  |  |
| 9 | When there is trouble, I can usually think of some way to deal with it. |  |  |  |  |
| 10 | No matter what happens to me, I can handle it. |  |  |  |  |
